# Structural, optical and vibrational properties of self-assembled Pb*_n_*_+1_(Ti_1−*x*_Fe*_x_*)*_n_*O_3*n*+1−*δ*_ Ruddlesden-Popper superstructures

**DOI:** 10.1038/srep07719

**Published:** 2015-01-16

**Authors:** K. I. Doig, J. J. P. Peters, S. Nawaz, D. Walker, M. Walker, M. R. Lees, R. Beanland, A. M. Sanchez, C. F. McConville, V. R. Palkar, J. Lloyd-Hughes

**Affiliations:** 1 University of Oxford, Department of Physics, Clarendon Laboratory, Parks Road, Oxford, OX1 3PU, United Kingdom; 2 University of Warwick, Department of Physics, Gibbet Hill Road, Coventry, CV4 7AL, United Kingdom; 3 Indian Institute of Technology Bombay, Mumbai 400076, India

## Abstract

Bulk crystals and thin films of PbTi_1−*x*_Fe*_x_*O_3−*δ*_ (PTFO) are multiferroic, exhibiting ferroelectricity and ferromagnetism at room temperature. Here we report that the Ruddlesden-Popper phase Pb*_n_*_+1_(Ti_1−*x*_Fe*_x_*)*_n_*O_3*n*+1−*δ*_ forms spontaneously during pulsed laser deposition of PTFO on LaAlO_3_ substrates. High-resolution transmission electron microscopy, x-ray diffraction and x-ray photoemission spectroscopy were utilised to perform a structural and compositional analysis, demonstrating that 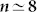 and 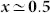. The complex dielectric function of the films was determined from far-infrared to ultraviolet energies using a combination of terahertz time-domain spectroscopy, Fourier transform spectroscopy, and spectroscopic ellipsometry. The simultaneous Raman and infrared activity of phonon modes and the observation of second harmonic generation establishes a non-centrosymmetric point group for Pb*_n_*_+1_(Ti_0.5_Fe_0.5_)*_n_*O_3*n*+1−*δ*_, a prerequisite for (but not proof of) ferroelectricity. No evidence of macroscopic ferromagnetism was found in SQUID magnetometry. The ultrafast optical response exhibited coherent magnon oscillations compatible with local magnetic order, and additionally was used to study photocarrier cooling on picosecond timescales. An optical gap smaller than that of BiFeO_3_ and long photocarrier lifetimes may make this system interesting as a ferroelectric photovoltaic.

In magnetoelectric multiferroics, where magnetisation and electrical polarisation are strongly coupled^1^,^2^,^3^,^4^, there is potential for application as capacitors, transducers and actuators^5^, and in magnetic data storage and spintronic devices^6^. Coupled ferroelectric and ferromagnetic orders provide additional possibilities for device design, such as magnetic control of ferroelectric polarisation (and vice versa), or the development of 4-state resistive memory^7^. Ferroelectric photovoltaics are an emerging area of interest as, unlike traditional semiconductor p-n junctions, photovoltages in ferroelectrics are not limited by their band gap^8^.

Oxides with the ABO_3_ perovskite structure are remarkably capable in accomodating defects, owing to their high dielectric constants and structural stability. While a small deviation from the 1:1:3 stoichiometric ratio produces point defects, larger deviations can lead to extended (planar) defect formation. Non-conservative crystallographic shear planes can account for oxygen deficiency^9^,^10^ in A*_n_*B*_n_*O_3*n*−2_, while the Ruddlesden-Popper (RP) homologous series A*_n_*_+1_B*_n_*O_3*n*+1_ has extra AO planes^11^. The unit cell of the RP phase consists in the *z*-direction of *n* ABO_3_ layers, a planar stacking fault [*a*/2, *b*/2, *d*] (where 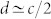), another *n* ABO_3_ layers and a second stacking fault. The epitaxial growth of strained RP phases can result in novel phases where *n* can be used to tune functional properties. For instance, ferroelectricity has been reported in the RP series Sr*_n_*_+1_Ti*_n_*O_3*n*+1_ only when *n* ≥ 4 and under tensile biaxial strain^12^. Conversely, out-of-phase boundaries (e.g. antiphase boundaries), where adjacent regions are offset by a fraction of a unit cell^13^, may be detrimental to long-range ferroic order.

Room temperature multiferroics are relatively rare, as common mechanisms for ferroelectricity and ferromagnetism are mutually exclusive: ferroelectricity generally requires empty d-orbitals, while ferromagnetic ordering often needs partial d-orbital occupancy^14^. Bismuth ferrite (BiFeO_3_) is one of the few room temperature multiferroics, and as such has been studied extensively^15^. Recently, PbTi_1−*x*_Fe*_x_*O_3−*δ*_ has been reported to be multiferroic at room temperature^16^. PbTiO_3_ is a displacive ferroelectric^17^ that exhibits a tetragonal bulk phase with space group P4mm^18^,^19^. Ferroelectric polarisation arises from the displacement of Ti^4+^ ions with respect to the surrounding oxygens. The partial substitution of Fe on the B site allows magnetism to be induced, but causes a reduction in the tetragonal distortion *c*/*a* and a concommitant decrease in the remnant polarization^18^,^20^,^21^,^22^. Similar to other multiferroics the use of thin films^23^ and nanoparticles^20^,^21^,^24^ may enhance ferroelectricity and ferromagnetism in comparison to the bulk phase. Negligible leakage current and unsaturated ferroelectric hysteresis loops have been reported for thin films^25^. Evidence of magneto-electric coupling in PTFO has been observed with electrically written domains visible in magnetic force microscopy images^23^.

In this article we report a study of the structural, optical and vibrational properties of compressively strained thin films of the Ruddlesden-Popper phase Pb*_n_*_+1_(Ti_0.5_Fe_0.5_)*_n_*O_3*n*+1−*δ*_ grown on LaAlO_3_ substrates. A structural characterization via X-ray diffraction and transmission electron microscopy (TEM) demonstrates the spontaneous formation of a Ruddlesden-Popper superstructure with 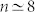, with a complex microstructure that exhibits a modulated atomic density in the growth direction. X-ray photoelectron spectroscopy (XPS) was used to determine the films' stoichiometry and chemical environments. The simultaneous Raman and infrared activity of phonon modes, along with the observation of second harmonic generation, identify a polar point group compatible with ferroelectricity. The dielectric function was extracted from the far-infrared to the UV using THz time-domain spectroscopy, Fourier transform infrared (FTIR) spectroscopy and UV-visible ellipsometry.

## Results

### Structure and composition

**Figure 1:**
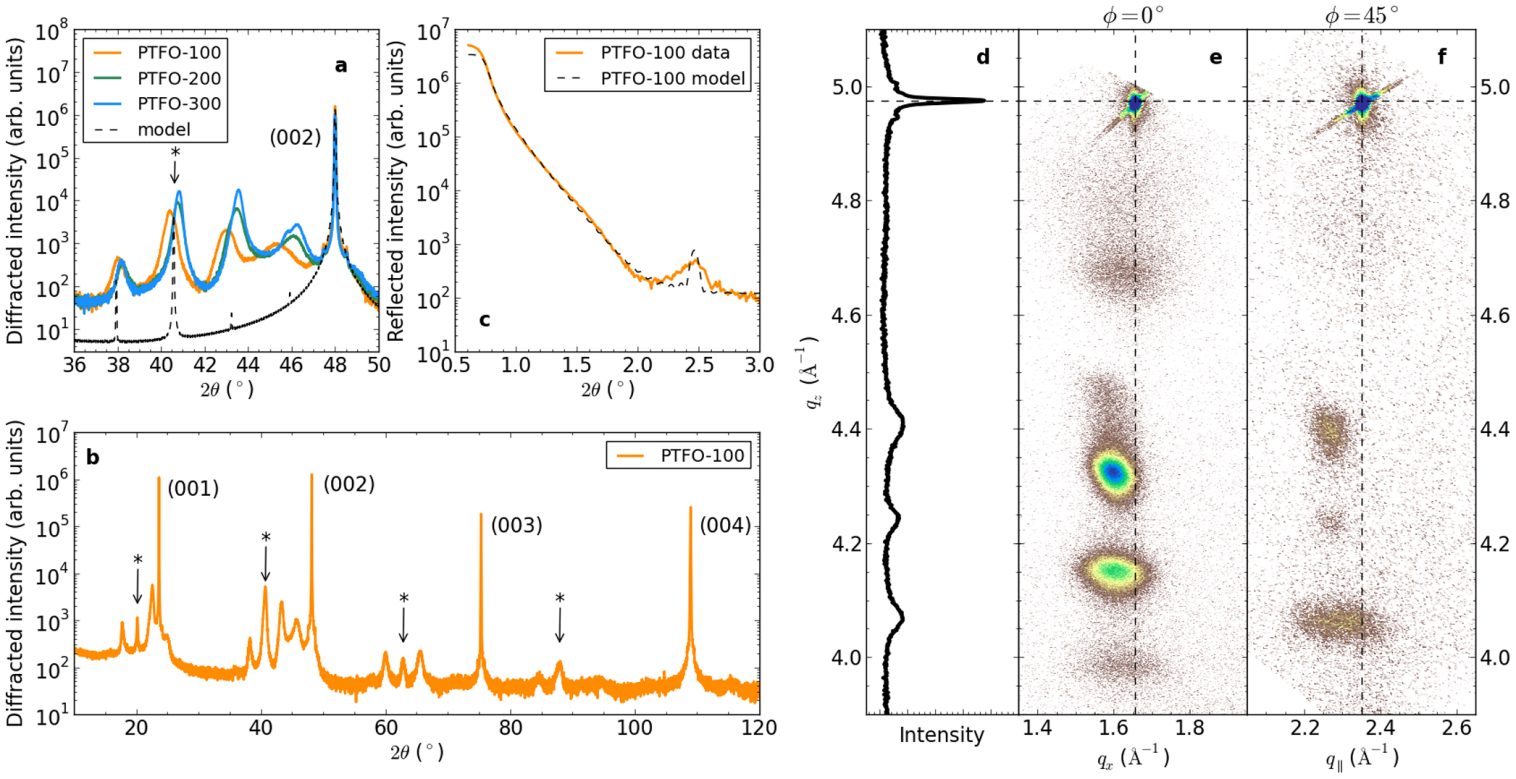
X-ray characterisation of Pb*_n_*_+1_(Ti_0.5_Fe_0.5_)*_n_*O_3*n*+1−*δ*_ films: (a) 2*θ* scans around the LaAlO_3_ substrate's (002) peak for PTFO-100, -200 and -300 films (solid lines). The asterisk marks the PTFO's Bragg peak corresponding to 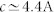. The dashed line is the model described in the text. (b) Wide angle 2*θ* scan for PTFO-100. (c) Shallow angle x-ray reflectivity for PTFO-100 from experiment (solid line) and simulation (dashed line). (d) A symmetric 2*θ* − *ω* scan for PTFO-100 around the (003) substrate peak, at *ϕ* = 0. (e), (f) Reciprocal space maps for PTFO-100 around the (103) and (113) LaAlO_3_ substrate peaks (indicated by the dashed lines), respectively. *q_x_*, *q*__∥__ and *q_z_* denote the scattering wavevectors in the [100], [110] and [001] directions of the substrate.

Thin films of PTFO with nominal thicknesses of 100 nm, 200 nm and 300 nm were deposited on (001) LaAlO_3_ (LAO) by pulsed laser deposition (see Methods), and are referred to herein as PTFO-100, PTFO-200 and PTFO-300. High-resolution 2*θ* − *ω* X-ray diffraction scans were taken to examine the crystal structure of the films. In Figure 1a the pseudocubic (002) peak of the LaAlO_3_ substrate (space group 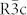, a = 3.789Å, *α* = 90.12°)^26^ is visible at 2*θ* = 48°. For each film a sequence of diffraction peaks at lower 2*θ* (larger *c*) than the substrate peak is evident. The wider range 2*θ* − *ω* scan in Figure 1b indicates diffraction peaks up to the substrate's (004) peak for the PTFO-100 sample. While the (002) film peaks are at lower 2*θ* than the LAO peak, those for (001) straddle the substrate peak (23.5°) with one of the four at a higher angle. The data resemble the diffraction pattern of a superlattice owing to their regular spacing in 2*θ*.

**Figure 2:**
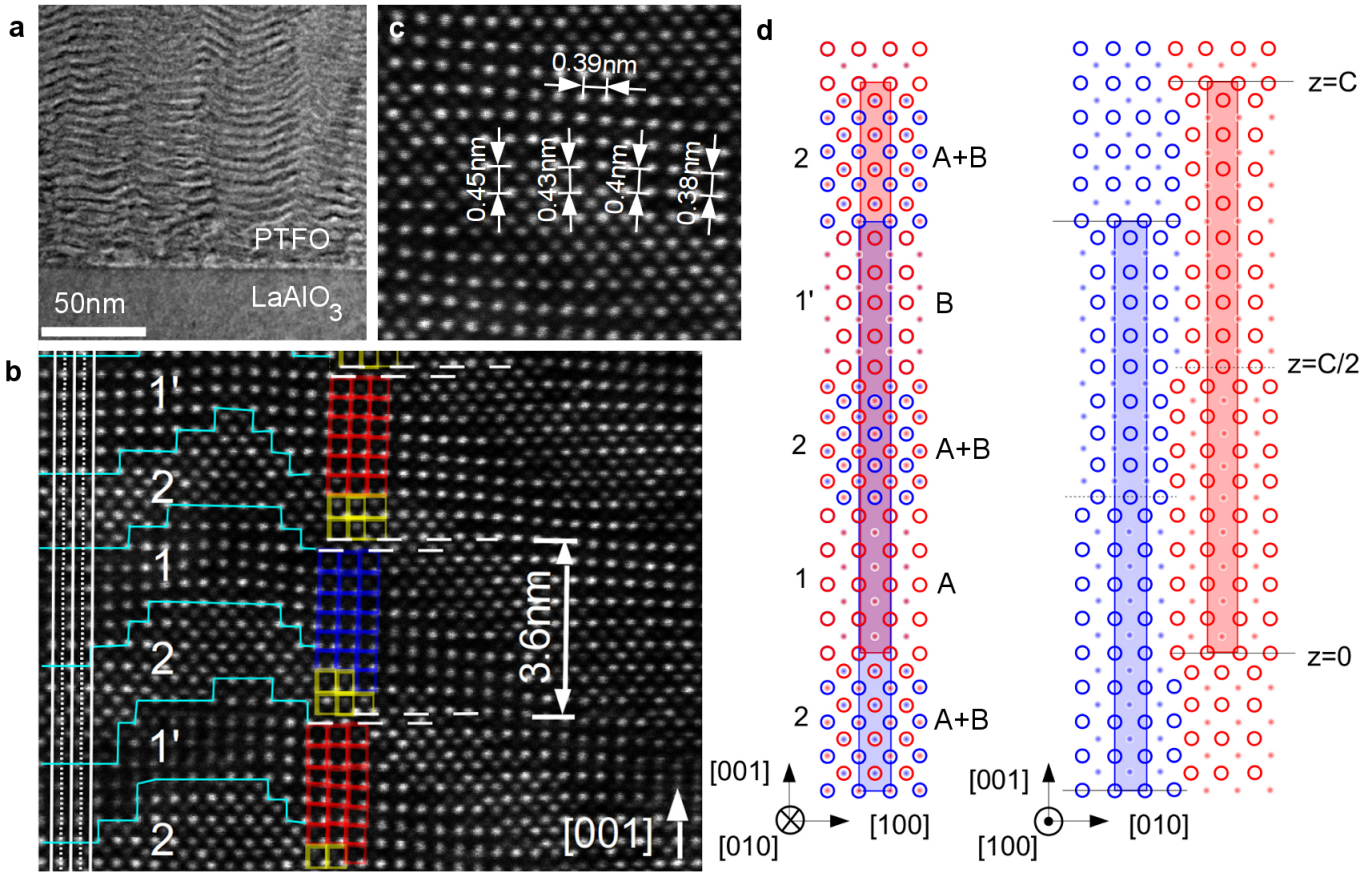
Transmission electron microscopy of 100 nm PTFO film. (a) Conventional TEM showing ‘wave' patterns. (b) ADF-STEM image showing that ‘waves' consist of a perovskite-like areas (labelled 1 and 1′, blue and red squares) and a rock-salt-like areas (labelled 2, yellow squares). Cyan lines mark the boundaries between these regions. The vertical white solid lines run through the perovskite A site in area 1, becoming the B site in area 1′. The horizontal white dashed lines show atomic planes separated by a stacking fault with 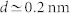. (c) ADF-STEM image showing a change in *c*, present in region 1 and 1′. (d) Schematic of structure in (010) plane (left, corresponding to plane of TEM image) and (100) plane (right). A Ruddlesden-Popper unit cell for *n* = 8 is shaded in blue, while another, offset by an antiphase boundary (see text), is shaded in red. Projection of the right-hand cartoon along [010] yields the left-hand schematic, creating areas where Pb ions appear to be on both the A and B sites.

Conventional TEM and ADF-STEM images (see Methods) of the atomic structure of the PTFO-100 film are reported in Fig. 2. Bright and dark wave-like patterns can be seen in the low-magnification image of Fig. 2a, with a periodicity in the growth direction of about 4 nm. The ADF image, showing the Pb columns most clearly (Ti atomic columns give weaker contrast), demonstrates that the “waves” consist of areas that appear perovskite-like (labelled 1 and 1′) and others that appear rock-salt-like (areas 2). The A- and B- cation site positions can be seen to reverse on opposite sides of the rock-salt-like layers: rather than lying on the solid white vertical lines in Fig. 2b, which run through the perovskite A-sites in area 1, the Pb atoms in areas 1′ are instead found on the dashed lines (B-sites of area 1). EELS spectra taken in areas 1, 2 and 1′ showed no difference in Fe or Ti composition (Supplementary Fig. S.1), implying that the different regions have similar stoichiometry. Further, TEM-EDX maps over regions up to 75 nm wide showed no variation in composition across each film (Supplementary Fig. S.2).

The structure can be understood as consisting of a RP phase with 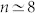 in the [001] growth direction, as pictured schematically in Fig. 2d. The left-hand panel pictures the (010) plane, corresponding to the plane of the TEM specimen, while the right-hand panel depicts the (100) plane. Two RP unit cells (dimensions *a*,*a*,*C*) are shaded in blue and red, where red atoms have been shifted by *C*/2 in the [001] direction (with respect to the blue atoms) by an antiphase boundary. Projecting the right-hand panel along the [010] direction results in the left-hand panel, which contains rock-salt-like regions (areas 2), as observed in TEM, with Pb ions on the A and B sites. The wave-like structures in Fig. 2a and b may result from either (a) a sharp rock-salt-like planar fault, lying on a plane inclined to the electron beam or (b) a diffuse fault with an extended rock-salt structure. Since TEM images are a projection of the three-dimensional structure it is hard to distinguish between these two possibilities.

Angle-dependent X-ray photoelectron spectroscopy (XPS) on the PTFO films identified two contributions to the Pb 4*f* peaks, as reported in Supplementary Fig. S.3a. Component peaks of a surface lead oxide, with enhanced contributions at a shallower take-off-angle, are present at 0.6–0.7 eV higher binding energy than the principal peaks from the perovskite-like PTFO film. As lead is volatile, lead oxide-rich surface regions can form spontaneously during PLD growth^27^. The observed binding energy is consistent with Pb_3_O_4_. Detailed fits to the XPS Fe 2p_3/2_, 2p_1/2_ and ‘shake-up' satellite spectra were consistent with Fe^3+^ ions (Supplementary Fig. S.3b).

The A:B:O ratio from XPS was (1.2 ± 0.3):1:(2.4 ± 0.3) relative to the B-site composition, after the contribution from the surface lead oxide was discluded. This is consistent with an oxygen-deficient RP phase in the limit of large *n*, when the RP phase tends to the ABO_3_ perovskite structure. Local EDX spectra yielded the composition Pb_1.75±0.18_Ti_0.53±0.05_Fe_0.5_O_3.44±0.34_ relative to Fe. The relative excess of Pb and O in the EDX composition may arise from lead oxide surface layers, which (unlike the XPS analysis) could not be resolved separately by EDX, and which may form during TEM specimen preparation. The Ti/Fe ratio determined from EDX was 1.06 ± 0.1, consistent with the 1:1 stoichiometry of the target. Considering ionic oxidation states only (Pb^2+^, Ti^4+^, Fe^3+^, O^2−^), charge balance requires that *δ* = 2 when *n* = 8 for Pb*_n_*_+1_(Ti_0.5_Fe_0.5_)*_n_*O_3*n*+1−*δ*_. An oxygen deficiency can be readily accomodated in oxide thin films by planar defects such as non-conservative boundaries^9^,^10^.

Returning to the XRD results, the position of each film's diffraction peaks can be assigned to the Bragg and superstructure peaks as follows. The * symbols in Fig. 1b denote the calculated Bragg angles for the PTFO-100 film assuming the perovskite cell has *c* = 4.437 Å. The adjacent satellite peaks are at angles consistent with a superlattice period Λ = 35.8 Å as determined using^28^: 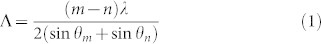
where *λ* = 1.540598 Å is the X-ray wavelength and *θ_m_* and *θ_n_* are the angular positions of adjacent satellite peaks, with orders *m* and *n*^29^. Table 1 lists *c* and Λ for the PTFO films assuming that *m* − *n* = 1. This results in 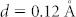, which is half the unit cell dimension *C* of the RP phase observed in TEM. The dashed line in Fig. 1a is a model calculation of the X-ray diffraction pattern of an RP phase on LAO (including Pb and La atoms only), assuming that *n* = 8, *c* = 4.43 Å and that the stacking fault had *d* = 0.12 Å. The model does not include disorder (such as variations in *c* or *n*), and hence has narrower diffraction peaks than observed experimentally. Figure 1d is a symmetric 2*θ* − *ω* scan for the PTFO-100 film around (003) at *ϕ* = 0, while panels e and f are reciprocal space maps around the (103) and (113) LAO substrate peaks respectively (indicated by the dashed lines). The RSMs show that the film's *a* = 3.90 Å lattice constant is slightly larger than that of the LAO substrate (*a* = 3.789 Å), and that there is negligible polycrystalline texture.

The ADF-STEM image of Fig. 2c indicates that areas of the perovskite-like layers 1 and 1′ have a reduced tetragonal distortion *c*/*a*. This changes the atomic density in these layers, creating a periodic modulation in the refractive index that can be seen in shallow-angle X-ray reflectivity scans [solid line in Fig. 1c]. The experimental data for the PTFO-100 sample exhibits a weak peak at 2*θ* = 2.45° indicative of a superlattice with period Λ = 37.6 Å (dashed line shows model, see Methods), consistent with the superlattice period calculated directly from the diffraction peaks (Table 1). Also evident in Fig. 2c are tilts of the local perovskite cell, which may result in the wave-like structure of Fig. 2a.

### Linear and non-linear optics in the visible-UV

**Figure 3:**
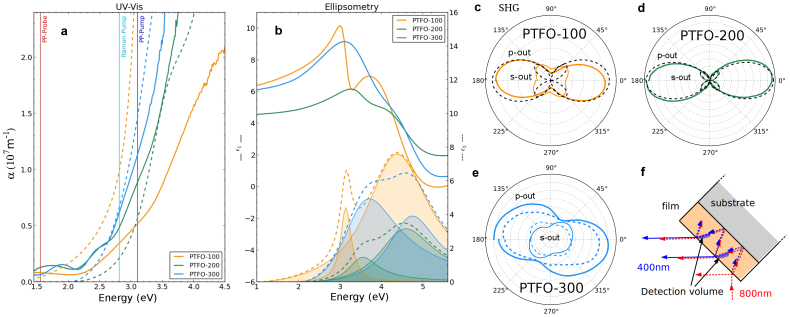
Linear and non-linear optics of PTFO films: (a) UV-visible absorption coefficient *α* from transmission at normal incidence (solid lines), and from ellipsometry at various angles of incidence (dashed lines). Vertical lines indicate pump and probe energies used for ultrafast Pump-Probe (PP) spectroscopy and pump energy for Raman. (b) Real (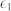, solid lines) and imaginary (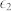, dashed) parts of complex dielectric function in the UV-visible range, as determined from ellipsometry. Shaded areas show the contribution of each mode to 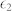. (c), (d) and (e) show the SHG radiation patterns for PTFO-100, -200 and -300 respectively, as a function of sample azimuthal angle *ϕ* (*ϕ* = 0 corresponding to p-polarised input) for *θ* = 45°. Data are shown for *p*-polarised (“*p*-out”) and *s*-polarised (“*s*-out”) detection. In (c) and (d) the dashed black lines show the SHG intensities expected for a tetragonal phase from the model described in the text. (f) SHG (blue) can only be detected from the “detection volumes” (blue shaded areas) defined by the film's finite absorption depth at the second harmonic.

The UV-visible absorption coefficient *α* at normal incidence and room temperature (see Methods) is reported in Figure 3(a). The absorption coefficient varies with the film thickness, and an increase in the absorption edge (~2.5 eV) is evident with reduced film thickness. The shift is most pronounced for the PTFO-100 film, and coincides with the increased tetragonal distortion for thinner films [Fig. 1(a) and Table 1]. In the region below the band gap, before the film begins to absorb strongly, the reported absorption coefficient exhibits oscillations that are artefacts caused by thin film interference.

The real and the imaginary part of the dielectric function (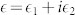) for PTFO-100, -200 and -300 extracted from ellipsometry measurements are shown in Fig. 3(b). The imaginary part was calculated using the Tauc-Lorentz model^30^, which has previously been applied to amorphous semiconductors^30^ and to the multiferroic BiFeO_3_^31^. The Tauc-Lorentz model accounts for a finite density of states below the bandgap, making it sensitive to the trap levels that play an important role in the bulk photovoltaic effect in ferroelectrics^8^,^32^,^33^.

Two Tauc-Lorentz oscillators with a common energy gap *E_g_* were used for each film. The determined film thicknesses were in good agreement with their nominal values, as summarised in Table 2, which also gives the parameters for the Tauc-Lorentz oscillators. Oscillators are represented graphically in Figure 3(b) by the shaded curves. As the film thickness is increased the central energy of the individual oscillators is blue-shifted. The dashed data in Figure 3(a) is the absorption coefficient as determined from ellipsometry. Note that no oscillatory artefact in the absorption is present below the band gap in the ellipsometry results (unlike *α* from UV-visible spectroscopy) because thin film interference is taken into consideration. Clear differences between the UV-visible transmission results (obtained at normal incidence) and the ellipsometry findings (at large angles) above the absorption edge may arise from the film's optical anisotropy in the growth (*z*) direction. A tetragonal phase will have a dielectric tensor with 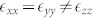, and this was not taken into account in the analysis of the ellipsometry data.

The second harmonic generation (SHG) intensity *I_p_*_,*s*_(*ϕ*) was obtained as a function of the polarisation angle *ϕ* of the incident 800 nm pump pulse (Methods), for *p*-polarised and *s*-polarised detection, as reported in Figs. 3(c)–(e) for the PTFO-100, -200 and -300 films. The clear SHG signal may arise from either (a) a non-centrosymmetric crystal structure at room temperature for the films, or (b) an interfacial contribution due to the break in translational symmetry (surface SHG). The peak SHG intensity *I_p_*(*ϕ*) was found to be in the ratio 1.0:8.0:0.7 for the PTFO-100, -200 and -300 films. The strong increase in SHG intensity between the PTFO-100 and PTFO-200 film indicates that any contribution from surface SHG is negligible in comparison to the SHG signal from the polar film, as a surface SHG contribution should not vary with film thickness. The observed intensity ratio can be qualitatively understood with reference to Fig. 3(f). The second harmonic (blue dashed lines) is generated throughout the film by the fundamental (red dashed lines) as the absorption coefficient at 800 nm is negligible. While the SHG intensity should increase with the length squared for a transparent medium, absorption in the films at 3.1 eV (400 nm) means that not all of the generated SHG is detected. Rather, the detected second harmonic comes from the region close to the film/air interface and for beams propagating in the correct direction, labelled the “detection volume” in Fig. 3(f). The depth of the detection volume can therefore be limited to the absorption depth, which is 1/*α* = 200 nm, 111 nm and 91 nm for the PTFO-100, -200 and -300 films [from Fig. 3(a)]. Thus, for the PTFO-100 film the SHG signal can come from the whole film thickness, while for the PTFO-300 film only the top 91 nm contributes. Since the thickest film exhibits smaller tetragonality *c*/*a* and strain relaxation in the detection volume (as evidenced by the XRD results), the SHG intensity is therefore reduced for PTFO-300.

The SHG *ϕ*-plots can be qualitatively understood as follows. Since the sample is rotated around the vertical axis by *θ* = 45° there is a maximum component of the electric field of the incident beam along the *c*-axis (spontaneous polarisation direction) for a *p*-polarised fundamental, leading to maxima in the SHG intensity at *ϕ* = 0 and *ϕ* = 180°, and a double-lobed shape. Quantitatively, for a tetragonal phase with point group 4 mm, the *ϕ*-dependence of the detected SHG intensity is *I_p_*(*ϕ*) = (*A* cos^2^ *ϕ* + *B* sin^2^ *ϕ*)^2^ and *I_s_*(*ϕ*) = *C* sin^2^ 2*ϕ*, where the constants *A*, *B* and *C* are linked to the SHG coefficients *d*_31_ and *d*_15_, as described by Kumar *et al.*^34^ for tetragonal-phase BiFeO_3_. The black dashed lines in Figs. 3(c)–(d) show such fits, which are in good agreement with experiment for the two thinner films. For the PTFO-300 *I_p_*_,*s*_(*ϕ*) varied somewhat between different spots on the sample [solid and dashed lines in Fig. 3(e)], and the maximum intensity was away from *p*-polarised incident light for *p*-polarised detection. The distortion of the crystal structure due to strain relaxation near the surface (in the detection volume), such that the spontaneous polarisation is no longer along [001], may explain shape of the *I_p_*_,*s*_(*ϕ*) for the PTFO-300 film. Similar results were obtained on polydomain 4 mm (Ba,Sr)TiO_3_ films with a deviation in ferroelectric polarisation from the *c*-axis^35^. Alternatively, anisotropy of the dielectric function at the second harmonic's wavelength may modify the polarisation state detected experimentally from the predicted pattern.

### Ultrafast optical response

**Figure 4:**
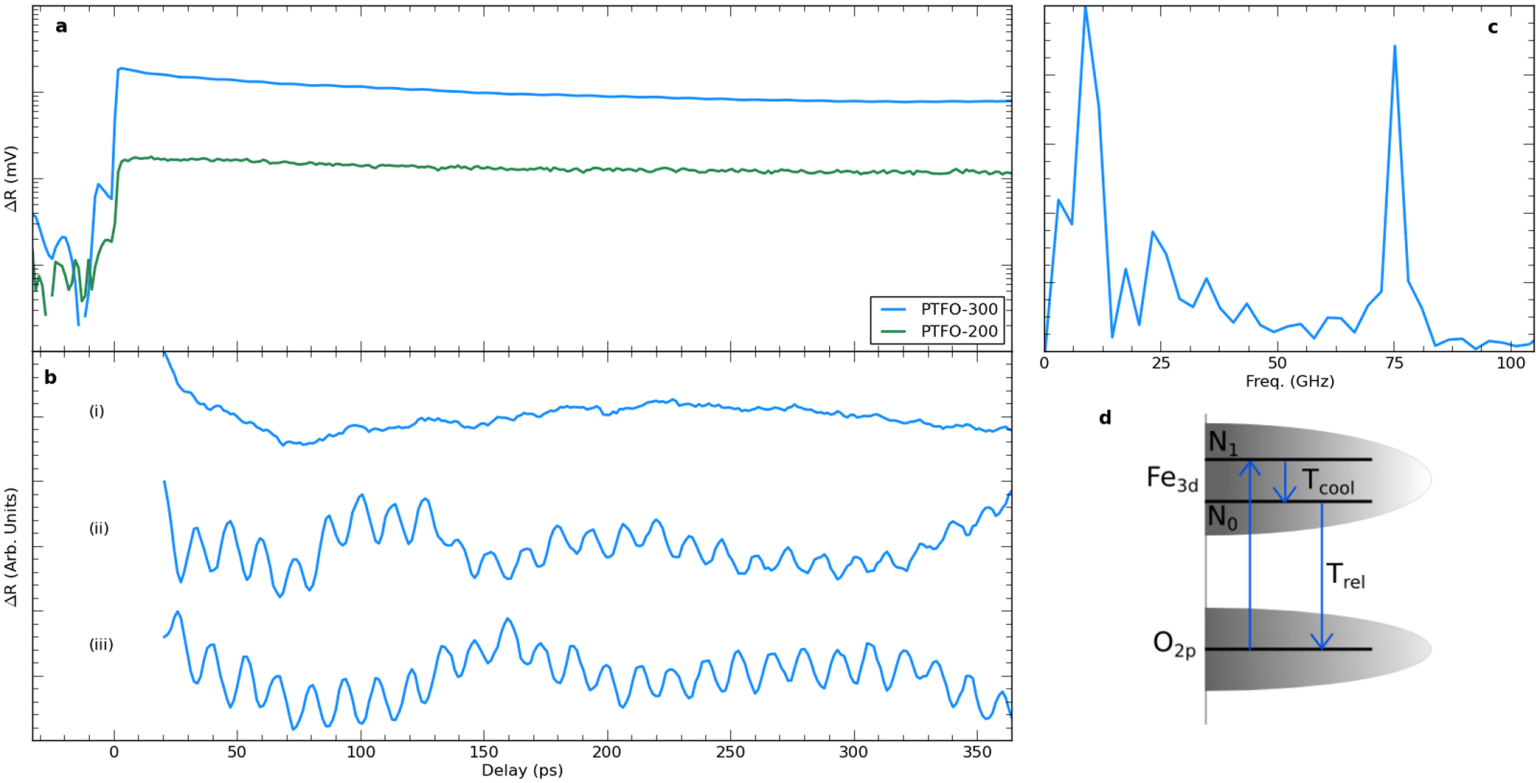
Pump probe spectroscopy of PbTiFeO_3_ films: (a) Raw pump probe for PTFO-300 (blue) and PTFO-200 (green). (b) Extracted oscillation for PTFO-300 with (i) no oscillation, (ii) oscillation and (iii) 180° phase shifted oscillation. (c) FFT of oscillation in b(ii). (d) Schematic diagram of carrier density model.

Photoinduced changes in reflectivity Δ*R* result from a modified refractive index Δ*n* (at the probe wavelength) caused by changes in the occupancy of electronic states. Time-resolved reflectivity thus permits electronic generation and relaxation processes to be examined. Further, coherent oscillations in Δ*R* can result from magnons in multiferroics^36^,^37^. The observed transients for the PTFO films (Fig. 4) comprise an electronic component with a sharp rise and a non-exponential decay for both films. This electronic component is described in more detail in the Discussion.

After numerical removal of the electonic contribution to Δ*R* a distinct oscillatory component superimposed on the reflectivity transient is observed for PTFO-300, as reported in Fig. 4(b), at a frequency of around 75 GHz [Fig. 4(c)]. The different curves (i)–(iii) correspond to different locations on the sample. While Δ*R* appeared as in curve (ii) in the majority of positions, in a smaller number of cases either no oscillations [curve (i)] or oscillations with a 180° phase shift [curve (iii)] were observed. Acoustic phonons can contribute to Δ*R* for oxide thin films^37^. The oscillation frequency depends upon the acoustic phonon velocity, and thus changes when the acoustic pulse propagates from the film to the substrate. Acoustic phonons in the substrate may create the peaks at low frequency (and from long time delays) in Fig. 4(c). However, the high frequency (75 GHz) feature cannot result from the acoustic phonon mechanism, as the oscillation is present at all time delays. In a previous study of magnons in BiFeO_3_ thin films a mode at a comparable frequency was found to disappear above the Néel temperature^37^. Both the PTFO films reported here and the BiFeO_3_ films previously studied^37^ had LaAlO_3_ substrates, and therefore have comparable in-plane lattice constants and Fe-Fe distances (assuming that there are regions of the PTFO samples with Fe ions in adjacent cells). It is thus plausible that the oscillation here is also a magnon. To confirm this, demonstrating that it disappears above the Néel temperature is desirable and will be the subject of further study. The occasional absence of the magnon and the 180° phase shift could be attributed to ferromagnetic domains or sample inhomogeneity.

### IR active phonons

**Figure 5:**
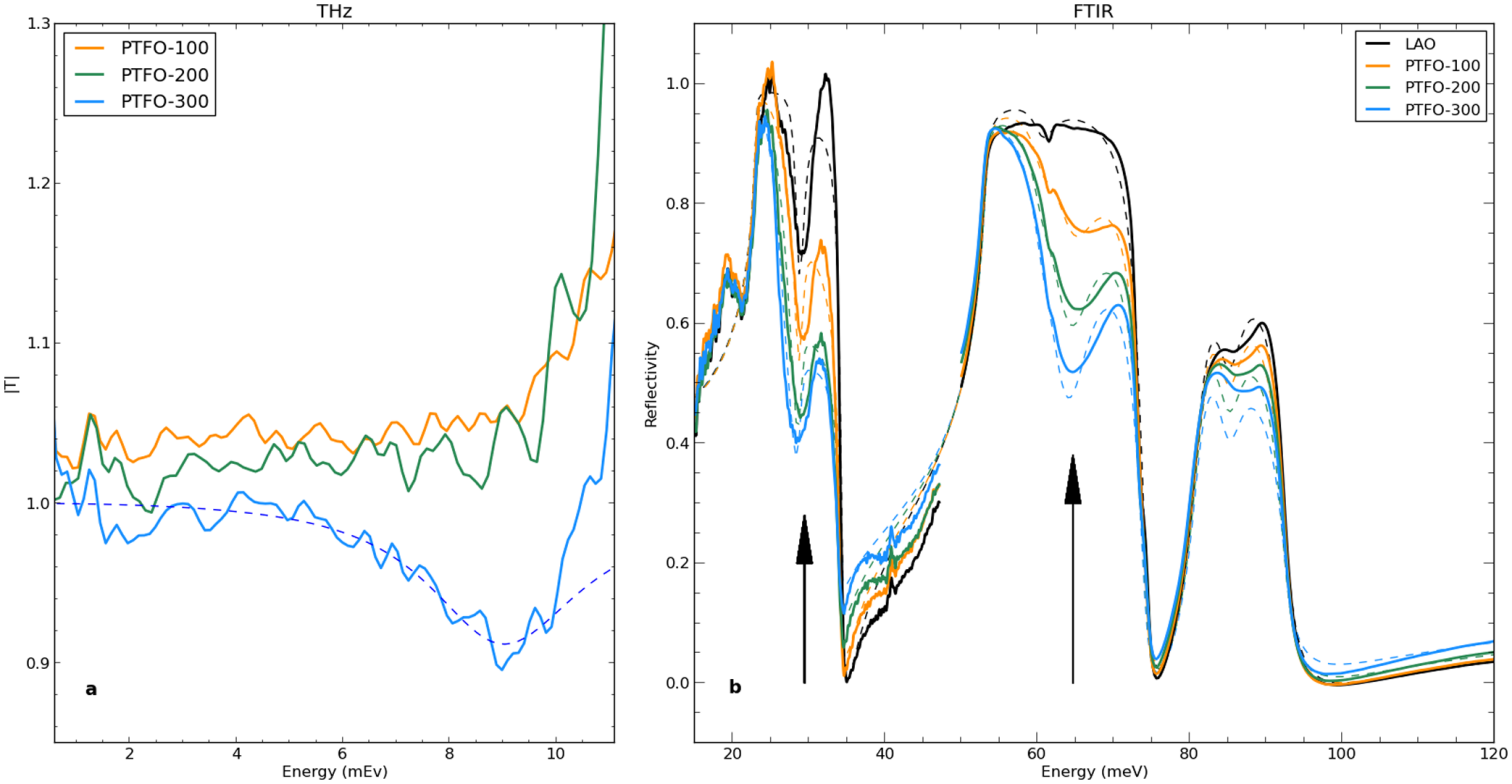
Infrared active vibrational modes in Pb*_n_*_+1_(Ti_0.5_Fe_0.5_)*_n_*O_3*n*+1−*δ*_ films: (a) Amplitude transmission |*T*| in the far-infrared (from THz-TDS, see Methods) relative to that through a bare LAO substrate. (b) Power reflectivity from FTIR spectroscopy (solid lines). Models (dashed lines) are described in the text.

Terahertz time domain spectroscopy^38^,^39^ was used to examine the dielectric function in the range 1–13 meV. The spectral transmission is reported in Fig. 5(a), and is flat and featureless for the PTFO-100 and PTFO-200 samples in the range below 10 meV. Above 10 meV all samples exhibit a sharp rise in transmission, an artifact which arises from the LaAlO_3_ substrate, which acts as a half wave plate at these frequencies^40^. The response for the PTFO-300 film displays a prominent reduction in transmission around 9 meV in Fig. 5(a). The dashed line indicates the transmission calculated for a thin film of PTFO on LaAlO_3_, assuming that the dielectric function of the film includes a phonon at 9.1 meV (2.2 THz) as described by Equation 2 (Methods) with linewidth Γ = 3.3 meV, 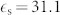 and 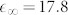. Here, 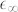 was chosen to match 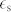 from the FTIR results.

FTIR reflectivity spectra are presented in Figure 5(b) (solid lines). The substrate data (black line) displays three Rehstrahlen bands. For the PTFO films the reflectivity lowers substantially in the areas marked by the arrows, and this reduction is more pronounced as the film thickness increases. This is a consequence of phonon modes in the film within the Rehstrahlen bands of the substrate. Reflectivity spectra were modelled (Methods) using two Drude-Lorentz oscillators for the films (parameters given in Table 2), and a 6-phonon model for the LaAlO_3_ substrate (similar to results reported elsewhere)^41^,^42^, producing the dashed lines. Oscillators in the PTFO films at 29.5 meV and 64.7 meV (see Table 2) are needed to create a good fit to experiment. There is little shift in the mode frequencies with thickness.

### Raman active phonons

**Figure 6:**
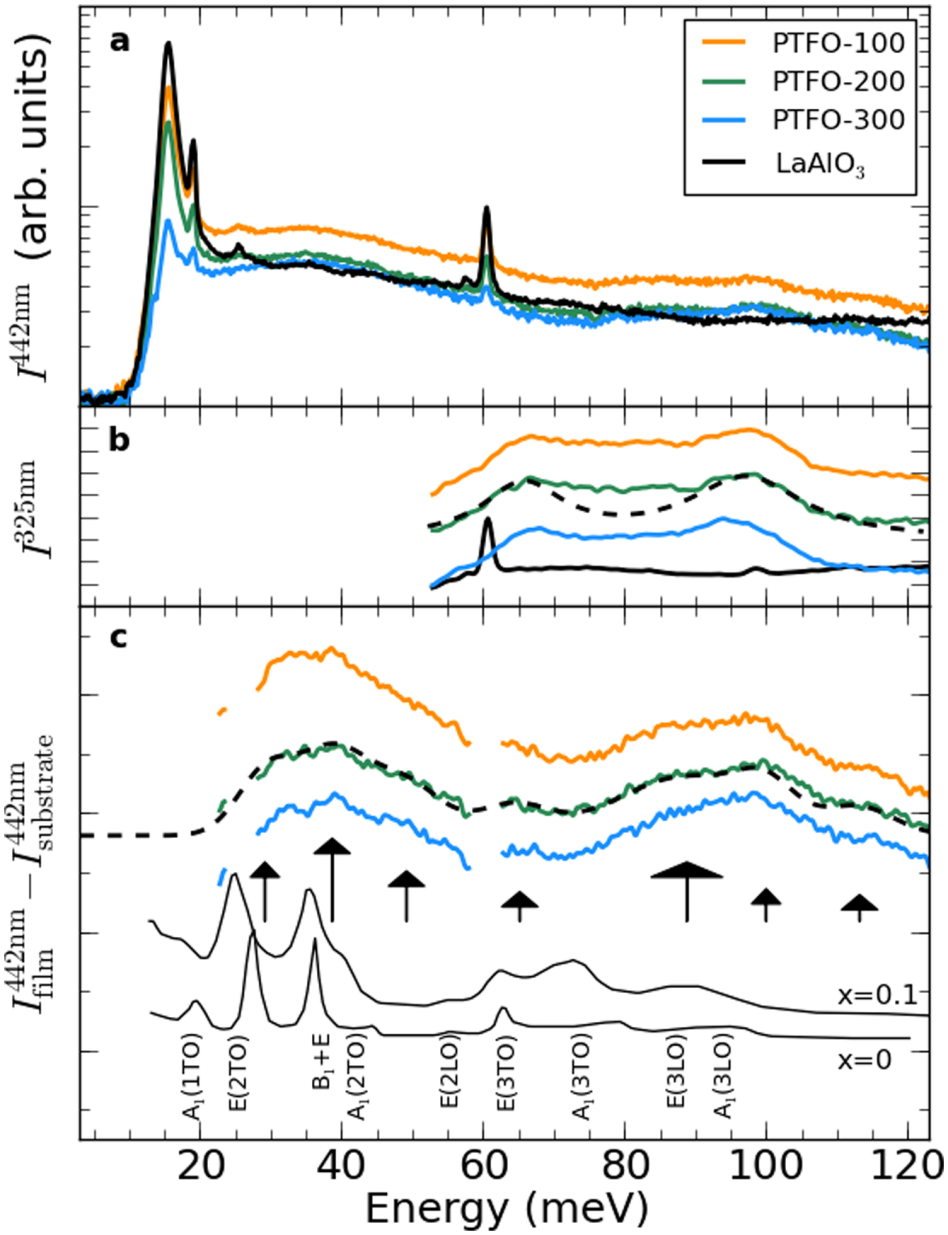
Raman spectra of PTFO films and LAO substrate: (a) Raman intensity *I* under 442 nm excitation. (b) *I* for 325 nm excitation. The dashed black line indicates a two-Lorentzian fit to the PTFO-200 spectra. (c) Raman intensity under 442 nm excitation after subtracting the substrate's contribution. The dashed black line is the model for PTFO-200 as described in the text and Table 2, where black arrows indicate individual oscillators. The lengths of the arrows represent the oscillator strengths, while the widths of the arrow heads denote the widths Γ. The thin back lines (bottom) are the data of Sun *et al.*^22^ for *x* = 0 and *x* = 0.1.

Raw Raman spectra for the PTFO samples and a (001)-oriented LaAlO_3_ reference sample obtained under 442 nm (2.81 eV) excitation are presented in Fig. 6(a). As the films are not strongly absorbing at this wavelength, there is a strong component from the LaAlO_3_ substrate (black line). In contrast, using 325 nm (3.82 eV) excitation, where the film absorbs strongly, yields a dominant Raman signal from the PTFO films [coloured lines in Figure 6(b)], but over a more limited spectral range (see Methods). For the data obtained at 442 nm the subtraction of the substrate's contribution^43^ permits the Raman spectra of the PTFO films to be seen [coloured lines in Figure 6(c)]. The narrow gaps correspond to the sharp Raman active modes 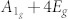 of the substrate, at 15.3 meV, 18.9 meV, 25.4 meV, 57.5 meV and 60.3 meV, corresponding well to previous reports^44^,^45^.

The Raman modes of the films are broader than those previously seen for bulk PTFO (with *x* = 0.05 and *x* = 0.1)^22^, which are reproduced by the solid thin black lines in Fig. 6(c) along with their mode assignments. Further, the Raman active modes of Pb*_n_*_+1_(Ti_0.5_Fe_0.5_)*_n_*O_3*n*+1−*δ*_ are shifted in energy in comparison to those at lower *x*^22^. The large changes in *c* evidenced by TEM will also alter the phonon eigenfrequencies, and this disorder will broaden the Raman resonances. As *a* does not vary substantially, eigenmodes that are IR-active in the plane may not be broadened as significantly. The PTFO-200 data was modelled by a series of Lorentzians (black dashed lines in panels b and c), where the arrows in Fig. 6(c) indicate the position and strength of the individual oscillators for the fits to the substrate-subtracted 442 nm data. For the fit in Fig. 6(b) [325 nm excitation, PTFO-200 film] only two Lorentzians were used, to better highlight the peaks at 65 meV and 97 meV.

Importantly, for ferroelectric media without an inversion centre vibrational modes can be both Raman and infrared active, as for PbTiO_3_^46^. For the two IR-active modes observed for Pb*_n_*_+1_(Ti_0.5_Fe_0.5_)*_n_*O_3*n*+1−*δ*_ at 29.5 meV and 64.7 meV in the FTIR results there are corresponding Raman active modes at 29 and 65 meV visible in the 442 nm Raman data [Figure 6(c)], and at 65 meV in the 325 nm Raman data [Figure 6(c)]. This discludes a centrosymmetric crystal structure for the Pb*_n_*_+1_(Ti_0.5_Fe_0.5_)*_n_*O_3*n*+1−*δ*_ films, as in a material possessing a center of symmetry no mode can be both infrared and Raman active by the rule of mutual exclusion.

## Discussion

Below, we contrast our results for the Ruddlesden-Popper phase Pb*_n_*_+1_(Ti_0.5_Fe_0.5_)*_n_*O_3*n*+1−*δ*_ with literature reports for perovskite phase PTFO and the model multiferroic BFO.

For PTFO powder samples a single perovskite phase is retained only for Fe fractions *x* ≤ 0.3, while *x* > 0.3 exhibits secondary phases visible as additional XRD peaks, and consisting mainly of PbFe_12_O_19_^18^. Here, the extra peaks visible in the XRD *ω* − 2*θ* scans arise from the RP superstructure, and no evidence of such secondary phases was observed via XRD or TEM.

To examine whether epitaxial strain or the oxygen-rich growth conditions are responsible for the formation of the RP phase it is desirable to examine films grown on different substrates. The deposition rate and substrate temperature can have a strong influence on the superlattice period, as reported for RP phases of barium stannate^47^. Crystallographic shear planes that lower oxygen content exhibit wave-like features and kinks in Pb_2_Sr_2_Bi_2_Fe_6_O_16_ crystals^10^, while 2D and 3D RP stacking faults have been witnessed in LaNiO_3_/LaAlO_3_ superlattices^48^ and Sr*_n_*_+1_Ti*_n_*O_3*n*+1_ ferroelectric films^12^.

In-plane and out-of-plane magnetisation measurements using a SQUID magnetometer (Methods) on the PTFO-300 film showed no evidence of ferromagnetic order for temperatures from 5 K to 300 K. Diamagnetic contributions from the LAO substrate and the sample holder were seen, as reported in Supplementary Fig. S.4. At low temperatures an increased magnetisation consistent with paramagnetic rare-earth impurity ions in the LAO substrate was observed. The lack of macroscopic ferromagnetic order contrasts with bulk PTFO and thin-films on alternative substrates, and may be a consequence of the spontaneous RP phase of these films. Alternatively, the disorder introduced by the variations in *c* and unit cell tilt throughout the film may disrupt magnetic order, as the strength of the superexchange interaction depends sensitively on Fe-O-Fe bond angles.

The direct optical absorption edge of the Pb*_n_*_+1_(Ti_0.5_Fe_0.5_)*_n_*O_3*n*+1−*δ*_ films, at about 2.5 eV, is comparable to that of rhombohedral BFO (2.7 eV) and below that of tetragonal BFO (3.1 eV)^31^. The onset of absorption, parameterised by the Tauc bandgap *E_g_*, is lower for Pb*_n_*_+1_(Ti_0.5_Fe_0.5_)*_n_*O_3*n*+1−*δ*_ (<2 eV) than BFO (2.14 eV and 2.30 eV for rhombohedral and tetragonal phase films)^31^. A detailed comparison between the UV-visible and ellipsometry results on Pb*_n_*_+1_(Ti_0.5_Fe_0.5_)*_n_*O_3*n*+1−*δ*_ reveals that there is a redshift in the absorption edge from UV-visible transmission for increasing film thickness (lower *c*), while the individual oscillator energies from ellipsometry blue shift. This can be understood as a consequence of the changes to oscillator widths Γ and amplitudes *A*: a slight increase in oscillator energies is swamped by a marked increase in Γ and *A* of the lowest mode. The increase in Γ could occur due to a larger variation in *c* throughout the thicker films.

Similar ultrafast reflectivity transients (with a rapid rise and a non-exponential decay) have been reported in recent studies of bulk BFO^49^,^50^ and thin films grown on a variety of substrates^37^,^51^,^52^. The rapid rise in reflectivity after excitation is attributed to the transfer of electrons between states. In BFO this is O_2*p*_ → Fe_3*d*_^53^; similar transitions to Fe_3*d*_ or Ti_3*d*_ are expected here. The electronic response for the PTFO-200 film is reduced in comparison to that of the PTFO-300 sample because of its reduced absorption at the pump energy [Fig. 3(a)].

The subsequent electronic decay can be approximated by the sum of two exponentials, 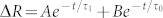, with lifetimes *τ*_1_ = 113 ps and *τ*_1_ = 78 ps for PTFO-200 and -300 respectively, and *τ*_0_ > 1 ns. However, such a biexponential model would signify that two independent photoexcited electron populations contribute to Δ*R*. As an alternative, a more physical model that includes carrier cooling between two levels^54^ was also found to describe the electronic behavior well. In this approach electrons are photoexcited with density *N*_1_ into an upper level 1, and relax with rate *N*_1_/*τ*_1_ (corresponding to the cooling rate of hot electrons) to lower level 0, as illustrated in the schematic in Fig. 4(d). This approach gave *τ*_1_ = 89 ps and *τ*_0_ = 2150 ps for PTFO-200, and *τ*_1_ = 63 ps, *τ*_0_ = 1350 ps for PTFO-300. The greater lifetime of the fast component for PTFO-200 can be explained by the closer proximity of the pump energy to the band edge, where cooling rates are lower. The *τ*_0_ > 1 ns lifetime for photocarrier recombination makes Pb*_n_*_+1_(Ti_0.5_Fe_0.5_)*_n_*O_3*n*+1−*δ*_ potentially useful in optoelectronic applications that require slow population decay, such as ferroelectric photovoltaics.

Large dielectric constants 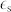 (reported here in the THz range) are often observed in ferroelectrics^55^ and incipient ferroelectrics. The extra mode seen in THz-TDS for PTFO-300 is not visible for the 100 nm or 200 nm samples. This may be a consequence of the experimental sensitivity, as thinner films produce smaller transmission changes, or be a feature of Pb*_n_*_+1_(Ti_0.5_Fe_0.5_)*_n_*O_3*n*+1−*δ*_ films above a certain thickness.

## Methods

### PLD

Pulsed laser deposition (PLD) was used to deposit films onto (001)-oriented LaAlO_3_ substrates. Target compositions were PbTi_1−*x*_Fe*_x_*O_3_ with *x* = 0.5. A laser fluence and frequency of 2 J/cm^2^ and 10 Hz, an oxygen pressure of 0.5 mbar, a substrate temperature of 625°C, and a substrate-target distance of 5 cm were employed. The deposition time was varied to get films with different thickness.

### TEM

Aberration-corrected transmission electron microscopy (TEM) was performed using an ARM200F system equipped with energy-dispersive X-ray (EDX) and electron energy loss spectroscopy (EELS) capabilities.

### XPS

An Omicron XM1000 monochromated Al K*_α_* x-ray source was used to illuminate the sample, with photoelectrons collected in an Omicron SPHERA analyser using an estimated sampling radius of 1.1 mm. Due to the insulating nature of the samples, an Omicron CN10 charge neutraliser was used to prevent surface charging. All binding energies were referenced to the C 1s peak from atmospheric contamination at 284.6 eV. All data were analysed using the CasaXPS package, with compositional analysis facilitated via determination of the analyser transmission function, calculated using polycrystalline Ag, Au and Cu foils.

### XRD

A four-circle x-ray diffractometer (PANalytical Xpert Pro MRD) with a Cu source and a 4-bounce hybrid monochromator was used to give pure K-*α*_1_ radiation (*λ* = 1.540598 Å). A Pixcel detector was used in scanning mode to collect the 2*θ* − *ω* diffraction scans. The reciprocal space maps were also collected in scanning mode as a collection of 2*θ* − *ω* scans, which were then converted into reciprocal space. Shallow-angle reflectivity data were modelled by the Genx software^56^ assuming the sample comprised a superlattice with two layers, one 2.2 nm layer (with *c* = 4.68 Å), and another 1.56 nm layer (*c* = 3.91 Å), with superlattice period 3.76 nm. The density in each bilayer was set by *c*.

### SQUID

The in-plane and out-of-plane magnetisation was measured with a SQUID magnetometer (Quantum Design MPMS-5S) from 5 K to 300 K in a magnetic field of up to *B* = 5 T.

### UV-visible transmission & ellipsometry

Room temperature transmittance spectra *T* were taken in the range 1–5 eV a using Perkin-Elmer LAMBDA 1050 UV/Vis/NIR Spectrophotometer at normal incidence. The absorption coefficient, *α* = −ln(*T*/*d*), was determined, where *d* is the film thickness from ellipsometry (see Table 2). Ellipsometry spectra (Δ, Ψ) for 3 angles of incidence (*θ* = 60°, 65° and 70°) were taken taken in the range 1–5.5 eV using a Horiba Scientific UVISEL Ellipsometer. The experimental spectra in Δ and Ψ were then fitted using a Tauc-Lorentz oscillator model^30^ to extract the dielectric function, 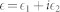. The Tauc-Lorentz model is made up of oscillators that share a common Tauc gap, *E_g_*. The real part of the dielectric function, 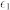 was calculated from the imaginary part, 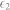 using the Kramers-Kronig relations.

### SHG and pump-probe reflectivity

A mode-locked Ti:sapphire laser oscillator (4 MHz, 650 nJ, 50 fs) provided the fundamental beam (800 nm or 1.55 eV) for SHG and time-resolved reflectivity. For SHG, a PMT placed after an analyser and short pass filters was used to detect the second harmonic. Samples were at *θ* = 45° from the normal, and with the incident beam polarised at angle *ϕ* (*ϕ* = 0 corresponding to p-polarisation). For the pump-probe reflectivity experiments the fundamental was doubled in frequency to 400 nm (3.1 eV) to photoexcite the multiferroic films at a fluence of 60 *μ*Jcm^−2^. The transient optical reflectivity subsequent to excitation was detected using a time-delayed fraction of fundamental beam, which was incident on the sample at close to normal incidence. The absorption depth of the samples for the pump and probe are 65–355 nm at *λ* = 400 nm and greater than 1.5 *μ*m at *λ* = 800 nm.

### Infrared and THz dielectric function

Terahertz time-domain spectroscopy^38^,^39^ was used to examine the dielectric function in the range 1–13 meV, from amplitude and phase resolved transmission measurements. The THz pulse was generated by a 800 nm, 20 fs pulse from a Ti:sapphire laser focused onto a GaAs photoconductive switch and detected by electro-optic sampling. The spectral transmission was calculated from the FFT of the time-domain data, using blank LaAlO_3_ as the reference. Data were averaged over four possible orientations of the PTFO samples and a blank substrate (VV, VH, HV, HH, where H/V represent horizontal or vertical orientation of the sample and substrate respectively). This removes the influence of substrate anisotropy^40^.

FTIR reflectivity spectra were taken using a Bruker Vertex 70v spectrometer at an angle of incidence of 11°. A globar light source was used, along with a KBr beamsplitter/DLaTGS detector for the mid-IR range (50–1000 meV), and a Si beamsplitter/DTGS detector for the far-IR range (15–48 meV). The RefFit software (http://optics.unige.ch/alexey/reffit.html) was used to model the reflectivity of thin film PTFO on LaAlO_3_. The Drude-Lorentz dielectric function was used, with the form: 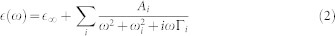
where 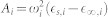 for each mode *i*.

### Raman spectroscopy

Raman spectra were taken using a Renishaw inVia Reflex Raman microscope with an excitation wavelength of 442 nm (2.81 eV) and focused with a 20× objective. UV-Raman spectra were taken under excitation at 325 nm (3.82 eV) and with a 40× objective. The Raman filters used for the 442 nm and 325 nm lasers cut out all signal below 10 meV and 52 meV, respectively, limiting the spectral range at low energies.

## Author Contributions

K.I.D. performed the XRD, optical, pump-probe and vibrational spectroscopy studies, aided by D.W. for the XRD experiments. J.L. and K.I.D. performed the SHG measurements. J.J.P.P., R.B. and A.M.S. undertook the TEM study. M.W. and C.F.M. performed and analysed the XPS results. M.L. performed the SQUID magnetometry. S.N. and V.R.P. grew the samples. K.I.D. and J.L. wrote the manuscript, and all authors discussed the results.

## Supplementary Material

Supplementary Information Supplementary Information

## Figures and Tables

**Table 1:** Summary of crystal structure of Pb*_n_*_+1_(Ti_0.5_Fe_0.5_)*_n_*O_3*n*+1−*δ*_ thin films from XRD

XRD	a (Å)	c (Å)	Λ (Å)
PTFO-100	3.90 ± 0.02	4.437 ± 0.009	35.8 ± 1.4
PTFO-200	3.90 ± 0.02	4.425 ± 0.005	36.1 ± 0.8
PTFO-300	3.90 ± 0.02	4.414 ± 0.005	35.6 ± 0.8

**Table 2:** Optical properties of Pb*_n_*_+1_(Ti_0.5_Fe_0.5_)*_n_*O_3*n*+1−*δ*_ thin films, from UV-visible ellipsometry (top), and FTIR spectroscopy/THz-TDS (bottom)

Ellipsometry		Oscillators				
	*E_i_* (eV)	*A_i_* (eV)	Γ*_i_* (eV)	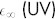	*E*_g_ (eV)	*δ* (nm)
PTFO-100	3.14	2.82	0.31	3.07	0.95	107
	4.42	22.76	1.85			
PTFO-200	3.51	6.65	0.88	3.3	1.97	225
	4.55	16.00	1.63			
PTFO-300	3.58	39.12	1.64	3.3	1.97	326
	4.72	16.73	1.45			
FTIR & THz-TDS		Oscillators				
	*E_i_* (meV)	*A_i_* (meV)	Γ*_i_* (meV)	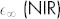		
PTFO-100	29.25	108.89	8.19	4.62		
	64.83	63.70	6.81			
						
PTFO-200	29.75	102.38	7.57	4.98		
	64.64	60.70	6.91			
						
PTFO-300	9.1	33.2	3.3	5.22		
	29.63	101.83	7.89			
	64.58	58.29	6.56			

## References

[b1] Kimura T. *et al.* Magnetic control of ferroelectric polarization. Nature 426, 55–58 (2003). 14603314 10.1038/nature02018

[b2] Wang J. *et al.* Epitaxial BiFeO_3_ multiferroic thin film heterostructures. Science 299, 1719–1722 (2003). 12637741 10.1126/science.1080615

[b3] Spaldin N. A. & Fiebig M. Materials science. The renaissance of magnetoelectric multiferroics. Science 309, 391–2 (2005). 16020720 10.1126/science.1113357

[b4] Ramesh R. & Spaldin N. A. Multiferroics: progress and prospects in thin films. Nat. Mater. 6, 21–9 (2007). 17199122 10.1038/nmat1805

[b5] Scott J. F. Applications of modern ferroelectrics. Science 315, 954–9 (2007). 17303745 10.1126/science.1129564

[b6] Bibes M. & Barthélémy A. Multiferroics: towards a magnetoelectric memory. Nat. Mater. 7, 425–6 (2008). 18497843 10.1038/nmat2189

[b7] Gajek M. *et al.* Tunnel junctions with multiferroic barriers. Nat. Mater. 6, 296–302 (2007). 17351615 10.1038/nmat1860

[b8] Bhatnagar A., Roy Chaudhuri A., Heon Kim Y., Hesse D. & Alexe M. Role of domain walls in the abnormal photovoltaic effect in BiFeO_3_. Nat. Commun. 4, 2835 (2013).

[b9] Abakumov A. M. *et al.* Slicing the perovskite structure with crystallographic shear planes: the A*_n_*B*_n_*O_3*n*−2_ homologous series. Inorg. Chem. 49, 9508–16 (2010). 20866030 10.1021/ic101233s

[b10] Batuk M. *et al.* Atomic structure of defects in anion-deficient perovskite-based ferrites with a crystallographic shear structure. Inorg. Chem. 53, 2171–80 (2014). 24479580 10.1021/ic4028404

[b11] Ruddlesden S. N. & Popper P. The compound Sr_3_Ti_2_O_7_ and its structure. Acta Cryst. 11, 54–55 (1958).

[b12] Lee C.-H. *et al.* Exploiting dimensionality and defect mitigation to create tunable microwave dielectrics. Nature 502, 532–6 (2013). 24132232 10.1038/nature12582

[b13] Zurbuchen M. *et al.* Morphology, structure, and nucleation of out-of-phase boundaries (OPBs) in epitaxial films of layered oxides. J. Mat. Res. 22, 1439–1471 (2007).

[b14] Hill N. A. Why are there so few magnetic ferroelectrics? J. Phys. Chem. B 104, 6694–6709 (2000).

[b15] Catalan G. & Scott J. F. Physics and Applications of Bismuth Ferrite. Adv. Mater. 21, 2463–2485 (2009).

[b16] Palkar V. & Malik S. Observation of magnetoelectric behavior at room temperature in Pb(Fe*_x_*Ti_1−*x*_)O_3_. Solid State Commun. 134, 783–786 (2005).

[b17] Cohen R. E. Origin of ferroelectricity in perovskite oxides. Nature 358, 136–138 (1992).

[b18] Ganegoda H., Kaduk J. A. & Segre C. U. X-ray powder diffraction refinement of PbTi_1−*x*_Fe*_x_*O_3−*δ*_ solid solution series. Powder Diffr. 28, 238–245 (2013).

[b19] Nelmes R. J. & Kuhs W. F. The crystal structure of tetragonal PbTiO_3_ at room temperature and at 700 K. Solid State Commun. 54, 721–723 (1985).

[b20] Ren Z. *et al.* Room-temperature ferromagnetism in Fe-doped PbTiO_3_ nanocrystals. Appl. Phys. Lett. 91, 063106 (2007).

[b21] Verma K. C., Kotnala R. K. & Negi N. S. Improved dielectric and ferromagnetic properties in Fe-doped PbTiO_3_ nanoparticles at room temperature. Appl. Phys. Lett. 92, 152902 (2008).

[b22] Sun C. *et al.* Negative thermal expansion in the PbTi_1−*x*_Fe*_x_*O_3_ system. Phys. Status Solidi B 245, 2520–2523 (2008).

[b23] Palkar V. R., Purandare S. C., Gohil S., John J. & Bhattacharya S. Scanning probe imaging of coexistent ferromagnetism and ferroelectricity at room temperature. Appl. Phys. Lett. 90, 172901 (2007).

[b24] Verma K. C., Kotnala R. K., Thakur N., Rangra V. S. & Negi N. S. Resistivity dependent dielectric and magnetic properties of Pb(Fe_0.012_Ti_0.988_)O_3_ nanoparticles. J. Appl. Phys. 104, 093908 (2008).

[b25] Sun C. *et al.* Ferroelectric and ferromagnetic properties of Pb(Ti_0.8_Fe_0.2_)O_3−*δ*_ thin film. Dalton Trans. 39, 9952–5 (2010). 20877818 10.1039/c0dt00681e

[b26] Hayward S. A. *et al.* Transformation processes in LaAlO_3_: Neutron diffraction, dielectric, thermal, optical, and Raman studies. Phys. Rev. B 72, 054110 (2005).

[b27] Vasco E., Bohme O., Roman E. & Zaldo C. Origin and control of the lead-enriched near-surface region of (Pb, La)TiO_3_. Appl. Phys. Lett. 78, 2037 (2001).

[b28] Fewster P. F. X-ray analysis of thin films and multilayers. Rep. Prog. Phys. 59, 1339–1407 (1996).

[b29] Fullerton E., Schuller I., Vanderstraeten H. & Bruynseraede Y. Structural refinement of superlattices from x-ray diffraction. Phys. Rev. B 45, 9292–9310 (1992). 10.1103/physrevb.45.9292 10000793

[b30] Jellison G. E. & Modine F. A. Parameterization of the optical functions of amorphous materials in the interband region. Appl. Phys. Lett. 69, 371 (1996).

[b31] Chen P. *et al.* Optical properties of quasi-tetragonal BiFeO_3_ thin films. Appl. Phys. Lett. 96, 131907 (2010).

[b32] Alexe M. Local mapping of generation and recombination lifetime in BiFeO_3_ single crystals by scanning probe photoinduced transient spectroscopy. Nano Lett. 12, 2193–8 (2012). 22468626 10.1021/nl300618e

[b33] Ruppel W., Von Baltz R. & Wurfel P. The origin of the photo-emf in ferroelectric and non-ferroelectric materials. Ferroelectrics 43, 109–123 (1982).

[b34] Kumar A. *et al.* Probing mixed tetragonal/rhombohedral-like monoclinic phases in strained bismuth ferrite films by optical second harmonic generation. Appl. Phys. Lett. 97 (2010).

[b35] Mishina E. D. *et al.* Domain orientation in ultrathin (Ba,Sr)TiO_3_ films measured by optical second harmonic generation. J. Appl. Phys. 93, 6216 (2003).

[b36] Talbayev D. *et al.* Detection of coherent magnons via ultrafast pump-probe reflectance spectroscopy in multiferroic Ba_0.6_Sr_1.4_Zn_2_Fe_12_O_22_. Phys. Rev. Lett. 101, 097603 (2008). 18851660 10.1103/PhysRevLett.101.097603

[b37] Doig K. I. *et al.* Coherent magnon and acoustic phonon dynamics in tetragonal and rare-earth-doped BiFeO_3_ multiferroic thin films. Phys. Rev. B 88, 094425 (2013).

[b38] Ulbricht R., Hendry E., Shan J., Heinz T. F. & Bonn M. Carrier dynamics in semiconductors studied with time-resolved terahertz spectroscopy. Rev. Mod. Phys. 83, 543–586 (2011).

[b39] Lloyd-Hughes J. & Jeon T.-I. A review of the terahertz conductivity of bulk and nanomaterials. J. Infrared Milli. Terahz. Waves 33, 871 (2012).

[b40] Lloyd-Hughes J., Jones S. P. P., Castro-Camus E., Doig K. I. & MacManus-Driscoll J. L. Modifying the polarization state of terahertz radiation using anisotropic twin-domains in LaAlO_3_. Opt. Lett. 39, 1121–1124 (2014). 24690686 10.1364/OL.39.001121

[b41] Calvani P. *et al.* Infrared optical properties of perovskite substrates for high-Tc superconducting films. Physica C 181, 289–295 (1991).

[b42] Shimada T., Kakimoto K.-I. & Ohsato H. Microwave dielectric properties of lanthanum aluminate ceramics and single crystal. J. European Ceram. Soc. 25, 2901–2905 (2005).

[b43] Iliev M. N., Abrashev M. V., Mazumdar D., Shelke V. & Gupta A. Polarized Raman spectroscopy of nearly tetragonal BiFeO_3_ thin films. Phys. Rev. B 82, 014107 (2010).

[b44] Abrashev M. V., Litvinchuk A. P., Iliev M. N. & Meng R. L. Comparative study of optical phonons in the rhombohedrally distorted perovskites LaAlO_3_ and LaMnO_3_. Phys. Rev. B 59, 4146–4153 (1999).

[b45] Saine M. C., Husson E., Brusset H. & Cerez A. Etude vibrationnelle d'aluminates et de gallates de terres rares-I. Alluminates de structure perovskite. Spectroc. Acta Pt. A-Molec. Biomolec. Spectr. 37, 985–990 (1981).

[b46] Foster C. M., Li Z., Grimsditch M., Chan S. K. & Lam D. J. Anharmonicity of the lowest-frequency A1(TO) phonon in PbTiO_3_. Phys. Rev. B 48, 160–167 (1993). 10.1103/physrevb.48.10160 10007291

[b47] Takahashi R. *et al.* Long-range spontaneous structural ordering in barium stannate thin films. Appl. Phys. Lett. 97, 081906 (2010).

[b48] Detemple E. *et al.* Ruddlesden-Popper faults in LaNiO_3_/LaAlO_3_ superlattices. J. Appl. Phys. 112, 013509 (2012).

[b49] Sheu Y. M. *et al.* Ultrafast carrier dynamics and radiative recombination in multiferroic BiFeO_3_. Appl. Phys. Lett. 100, 242904 (2012).

[b50] Ruello P. *et al.* Photoexcitation of gigahertz longitudinal and shear acoustic waves in BiFeO_3_ multiferroic single crystal. Appl. Phys. Lett. 100, 212906 (2012).

[b51] Jin Z. M. *et al.* Structural dependent ultrafast electron-phonon coupling in multiferroic BiFeO_3_ films. Appl. Phys. Lett. 100, 071105 (2012).

[b52] Chen L. Y. *et al.* Ultrafast photoinduced mechanical strain in epitaxial BiFeO_3_ thin films. Appl. Phys. Lett. 101, 041902 (2012).

[b53] Pisarev R., Moskvin A., Kalashnikova A. & Rasing T. Charge transfer transitions in multiferroic BiFeO_3_ and related ferrite insulators. Phys. Rev. B 79, 1–16 (2009).

[b54] Jepsen P. U. *et al.* Ultrafast carrier trapping in microcrystalline silicon observed in optical pump-terahertz probe measurements. Appl. Phys. Lett. 79, 1291 (2001).

[b55] Merz E. F. & Walter J. Ferroelectricity (North Holland Pub. Co., Amsterdam, 1967).

[b56] Björck M. & Andersson G. GenX: an extensible X-ray reflectivity refinement program utilizing differential evolution. J. Appl. Cryst. 40, 1174–1178 (2007).

