# Supplementary material for: Structural, optical and vibrational properties of self-assembled Pbn+1(Ti1−xFex)nO3n+1−δ Ruddlesden-Popper superstructures
Source: Sci Rep. 2015 Jan 16;5:7719. doi: 10.1038/srep07719 (PMC4296293; doi:10.1038/srep07719)
Supplement: Supplementary Information [file srep07719-s1.pdf]

Supplementary Information for: Structural, optical and vibrational properties of self-assembled $\mathbf{Pb}_{n+1}(\mathbf{Ti}_{1-x}\mathbf{Fe}_x)_n\mathbf{O}_{3n+1-\delta}$ Ruddlesden-Popper superstructures

K. I. Doig,[1] J. J. P. Peters,[2] S. Nawaz,[3] D. Walker,[2] M. Walker,[2] M. R. Lees,[2] R. Beanland,[2] A. M. Sanchez,[2] C. F. McConville,[2] V. R. Palkar,[3] and J. Lloyd-Hughes[2,*]

[1] *University of Oxford, Department of Physics, Clarendon Laboratory, Parks Road, Oxford, OX1 3PU, United Kingdom.*

[2] *University of Warwick, Department of Physics, Gibbet Hill Road, Coventry, CV4 7AL, United Kingdom*

[3] *Indian Institute of Technology Bombay, Mumbai 400076, India*

Abstract

In this Supplementary Information document we provide in Supplementary Figures S1 and S2 electron energy loss spectroscopy (EELS) and energy-dispersive X-ray (EDX) results from transmission electron microscopy. Supplementary Figure S3 provides a summary of X-ray photoemission (XPS) results. Magnetisation loops and the temperature-dependent susceptibility of a PTFO film on $LaAlO_3$, and a reference $LaAlO_3$ substrate, are reported in Supplementary Figure S4.

---

* J.Lloyd-Hughes@warwick.ac.uk

[1] L. A. J. Garvie and P. R. Buseck, **396**, 667 (1998), ISSN 0028-0836.

[2] A. Torres-Pardo, A. Gloter, P. Zubko, N. Jecklin, C. Lichtensteiger, C. Colliex, J.-M. Triscone, and O. Stéphan, Physical Review B **84**, 220102 (2011), ISSN 1098-0121.

[3] C. J. Lu, A. X. Kuang, and G. Y. Huang, Journal of Applied Physics **80**, 202 (1996), ISSN 00218979.

[4] M. C. Biesinger, B. P. Payne, A. P. Grosvenor, L. W. Lau, A. R. Gerson, and R. S. Smart, Applied Surface Science **257**, 2717 (2011), ISSN 01694332.

[5] C. Brundle, T. Chuang, and K. Wandelt, Surface Science **68**, 459 (1977), ISSN 00396028.

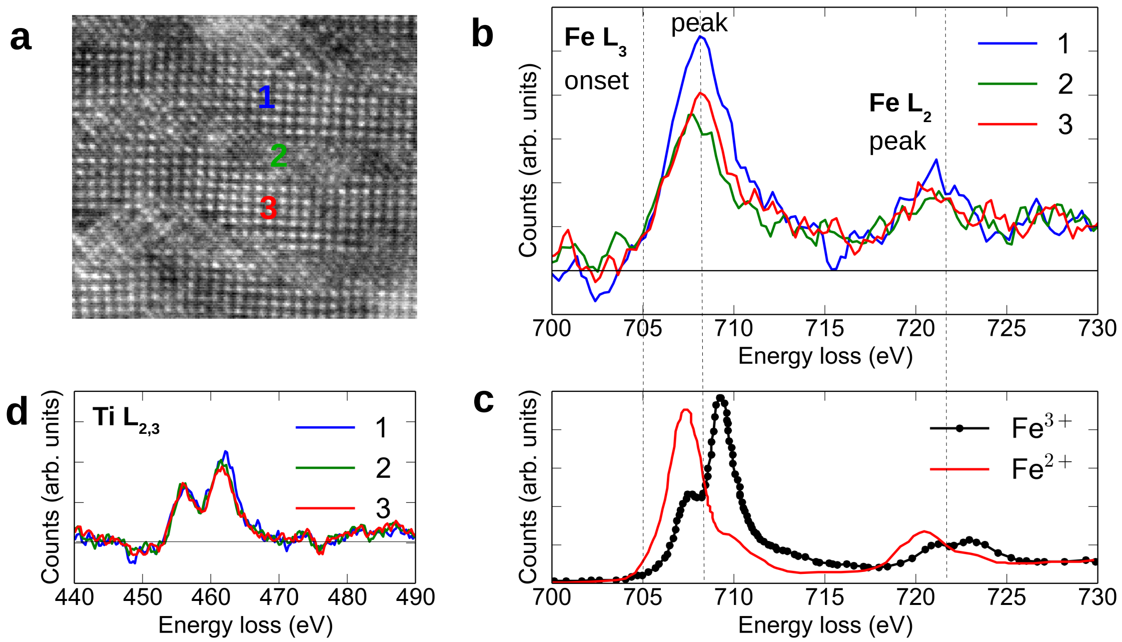

FIG. S.1. **a** HAADF image of PTFO. **b** Electron energy loss spectroscopy (EELS) of the Fe L_3 ($2p_{3/2} \rightarrow 3d$) and L_2 ($2p_{1/2} \rightarrow 3d$) edges of regions 1, 2 and 3 in **a**. There is no discernible variation in Fe content or environment between the regions. **c** Literature EELS Fe L_3 and L_2 spectra for Fe^{2+} and Fe^{3+} compounds [Data replotted from Ref. 1]. For the Ruddlesden-Popper phase PTFO films studied here, the Fe L_3 onset energy and peak energy are above that of Fe^{2+} and below that of Fe^{3+}, as illustrated by the dashed vertical lines in panels **b** and **c**. **d** The Ti $L_{2,3}$ EELS spectra also show no change in the different regions. The variation in unit cell size and shape of PTFO, evident in the TEM images (Figure 2 of main manuscript), may account for the broadened spectra in **b** and **d** in comparison to the literature,[1,2] since EELS $L_{2,3}$ spectra are highly sensitive to structural distortions.[2]

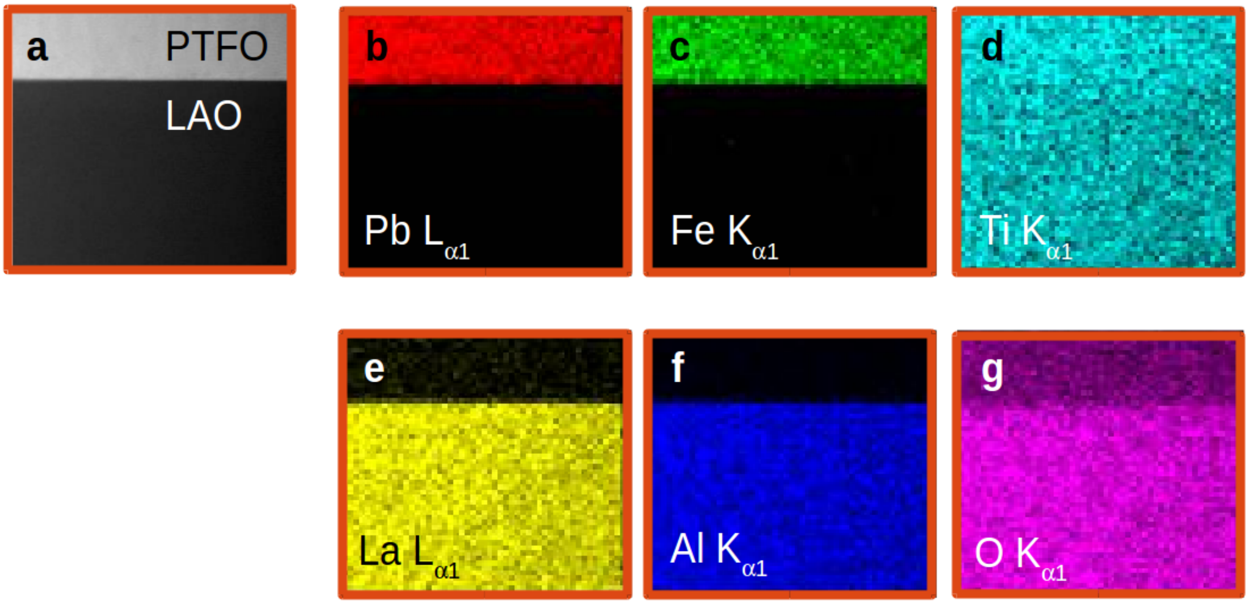

FIG. S.2. **a** Transmission electron microscopy (TEM) image and **b-g** energy-dispersive X-ray (EDX) spectroscopy maps of a PTFO/LAO interface. The boxes are about 75 nm × 75 nm. Pb, Fe, La and Al maps show a clear separation between the PTFO layer and the LAO substrate. The lack of contrast in the Ti map (and apparent contrast in the O map) is an artefact created by these peaks overlapping in energy. For the semi-quantitative analysis multiple least squares fits were used to obtain the atomic ratios, taking proper account of peak overlaps. The composition determined for the LAO substrate was $La_1Al_{1.08}O_{3.39}$, and hence a 10 % error in composition from EDX is stated in the main text.

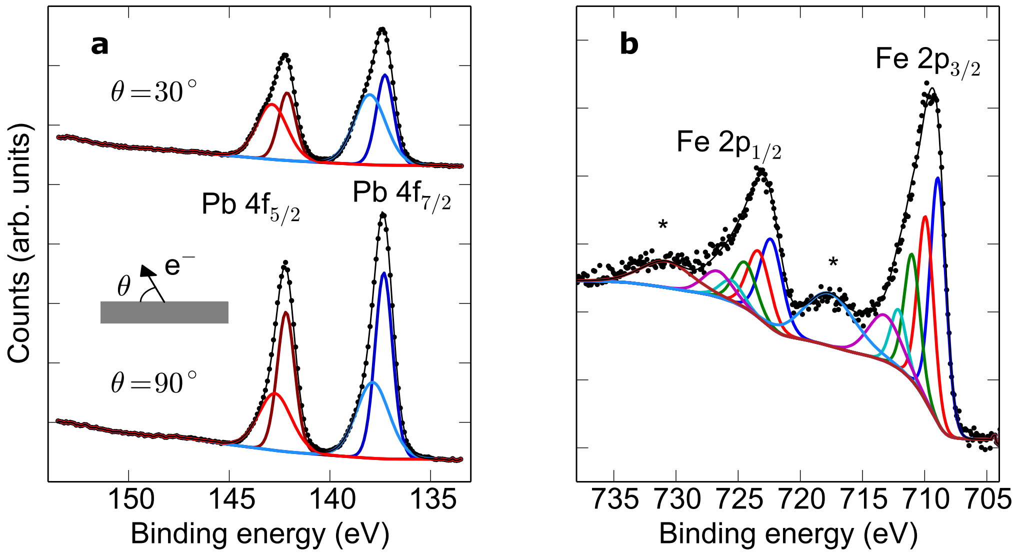

FIG. S.3. **a** XPS Pb 4f spectra at take-off-angle $\theta = 30°$ (top) and $90°$ (bottom) for PTFO-100. Points are experimental data. Two peaks are required to fit the Pb $4f_{7/2}$ (blue, 137.3 eV; light blue, 138.0 eV) and Pb $4f_{5/2}$ (dark red, 142.2 eV; red, 142.8 eV) regions. The overall fit is shown by a thin black line, and includes a Shirley background. At grazing emission the electron escape depth is reduced and XPS spectra represent electrons emitted from closer to the surface. As a result the relative area of the lower binding energy (BE) component reduces with respect to the higher BE component, indicating that the 137.3 eV and 142.2 eV peaks result from Pb in the PTFO thin film. By contrast, the relative increase in weight of the 138.0 eV and 142.8 eV peaks suggest Pb exisiting in a surface oxide layer. The binding energies of these contributions are consistent with Pb_3O_4. The Ti 2*p* spectra (not shown) exhibit a single binding energy comparable to that of $PbTiO_3$.[3]
b XPS spectra of the Fe 2p at normal emission $\theta = 90°$. Fits are shown (coloured lines) to the Fe $2p_{3/2}$ and $2p_{1/2}$ regions using the multiple reference samples with differing Voigt functions of Ref. 4 for Fe^{3+} ions in Fe_3O_4, but shifted downwards in BE by 1.2 eV. Each principal peak consists of 5 components with systematically decreasing amplitudes at higher binding energies.[4] The satellite peaks (marked $*$) are at $\Delta = 8.4 \pm 0.2$ eV (averaged over all data sets) higher than the principal ($2p_{3/2}$ or $2p_{1/2}$) peaks, consistent with Fe^{3+} ions for which $\Delta = 8.5$ eV.[5] In contrast, for Fe^{2+} a further peak would be expected at 1.8 eV lower BE than the first component shown. This is not observed.

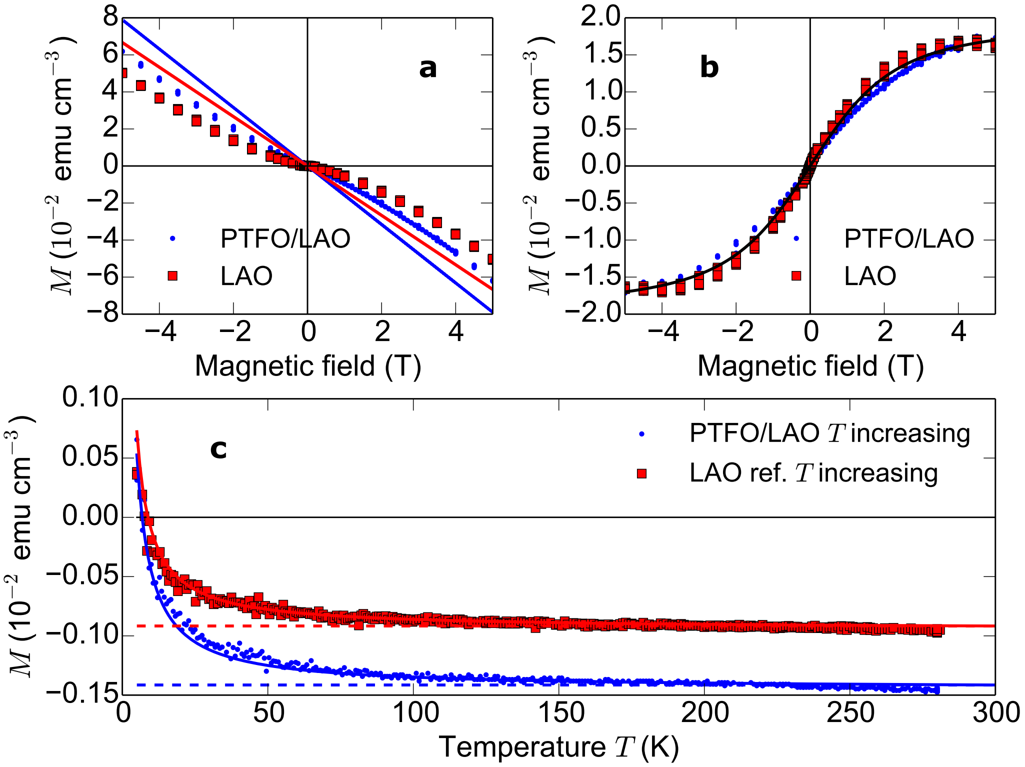

FIG. S.4. **a** Magnetisation M versus applied magnetic field ($B \parallel [100]$) at a temperature $T = 5$ K for the 300 nm-thick PTFO film on $LaAlO_3$ (blue points), compared with that from a reference $LaAlO_3$ substrate (red squares). Magnetisation loops for ($B \parallel [001]$) were similar. Straight lines indicate the diamagnetic contribution from the substrate and sample holder. **b** Magnetisation loops from **a** after subtraction of the diamagnetic (linear) component do not show significant hysteresis. The solid line shows the expected $M(B)$ for a paramagnet (Brillouin function with $J = 7/2$, scaled to fit the experimental data). **c** Magnetic moment at fixed applied magnetic field (0.1 T), (proportional to the susceptibility χ), versus temperature. At high temperature T the moments (and χ) tend to constant negative values (dashed lines, diamagnetism of substrate and sample holder). At low T an increase in moment (and thus χ) proportional to $1/T$ (solid lines) indicates paramagnetism. This is likely to be from rare-earth impurities in the $LaAlO_3$ substrate as both substrate and film show similar dependences. Large values of $J = 7/2$ are typically found for rare-earth ions with partially filled 4f orbitals (e.g. Gd^{3+}, Yb^{3+}).
